# Insights into the Controlled Formation of Zr‐Based Metal–Organic Gels: Linking Macroscopic Properties with Molecular Information from Solution State NMR

**DOI:** 10.1002/anie.202520987

**Published:** 2026-01-19

**Authors:** Juan C. Muñoz‐García, Francisco G. Moscoso, Elena M. Sánchez‐Fernández, Jenifer Santos, Jesús Angulo, Carolina Carrillo‐Carrión

**Affiliations:** ^1^ Institute for Chemical Research (IIQ) CSIC–University of Seville Seville 41092 Spain; ^2^ Center for Nanoscience and Sustainable Technologies (CNATS) Department of Physical, Chemical and Natural Systems, Pablo de Olavide University Seville 41013 Spain; ^3^ Department of Organic Chemistry Faculty of Chemistry University of Seville Seville 41012 Spain; ^4^ Department of Health and Biomedical Sciences Faculty of Health Sciences Universidad Loyola Andalucía Dos Hermanas Seville 41704 Spain

**Keywords:** Carbohydrate‐based drugs, Metal–organic gels, NMR spectroscopy, Spin diffusion transfer difference NMR, Water structuration

## Abstract

Understanding and controlling the formation mechanisms of metal–organic gels is crucial for their rational design with well‐defined properties for diverse applications. However, rapid methodologies enabling atomic‐resolution structural characterization of gel formation are still largely lacking. Here, we report for the first time the molecular‐level characterization of the in‐situ formation of a Zr‐based metal–organic gel by monitoring solvent structuration during gelation using solvent‐observed nuclear magnetic resonance (NMR) spectroscopy. UiO‐66‐type gels were optimized under mild conditions, i.e., 40 °C and in the absence of acidic modulators, providing a biocompatible environment suitable for the in‐situ encapsulation of sensitive biomolecules during gelation. The combined analysis of saturation transfer difference and spin diffusion transfer difference NMR growth curves enabled real time monitoring of nucleation and gelation stages, revealing an excellent correlation between the progressive structuration of water within the gel network and the resulting macroscopic properties. Furthermore, we demonstrate that this NMR approach allows tracking of the in‐situ encapsulation of therapeutic biomolecules within the gel, exemplified by a glycolipid with anti‐inflammatory properties.

## Graphical abstract

Real time STD/SDTD NMR unveils water structuring during UiO‐66 gelation under mild, acid‐free conditions compatible with biomolecule encapsulation. This approach bridges molecular‐scale solvent ordering with macroscopic gel properties, unlocking mechanistic insight for the rational design of MOF gels.

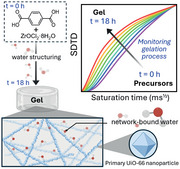

## Introduction

The exceptional level of tunability of metal–organic frameworks (MOFs), formed by polydentate organic ligands coordinated to metal‐containing nodes, has been key to positioning them as frontrunners among porous materials over the past decade.^[^ ^1^ ^]^ Many efforts have focused on achieving MOFs with varying molecular compositions, topologies, crystal structures, pore structures, morphologies, and surface functionalizations, allowing fine tuning of their properties and consequently enabling diverse applications, e.g., catalysis, gas adsorption, molecular separation, energy storage, sensing, and drug delivery.^[^ ^2^, ^3^ ^]^ Although the vast majority of MOFs synthesized so far are polydisperse microcrystalline powders, their ability to gelate under specific synthetic conditions opens new opportunities for preparing and shaping hierarchically porous MOF gels or monoliths.^[^ ^4^ ^]^ These forms exhibit distinctive properties not found in powdered form, such as higher packing density, enhanced mass transfer, and improved mechanical stability.^[^ ^5^, ^6^, ^7^ ^]^

By definition, according to the IUPAC,^[^ ^8^ ^]^ a MOF gel is a type of metal–organic gel (MOG), consisting of a colloidal network of discrete crystalline nanoparticles that aggregate via weak non‐covalent interactions throughout a liquid phase.^[^ ^4^ ^]^ Compared to bulk crystalline MOFs, a MOF gel features porosity across at least two different length‐scales, thanks to combining the intrinsic micro‐/meso‐porosity of the MOF structure (i.e., well‐ordered intraparticle pores) with meso‐/macro‐porosity derived from the gel state (i.e., interparticle pores). To obtain a MOF gel, experimental conditions must be precisely optimized in order to avoid the formation of microcrystalline powder products. Understanding the crystallization process in MOFs is therefore critical. In this context, Bennett et al.^[^ ^4^ ^]^ brilliantly summarized mechanistic aspects of MOF gel formation, emphasizing that the proper balance between nucleation and crystal growth rates is essential for promoting gel formation. Importantly, these two processes, nucleation and crystal growth, can be influenced by synthetic parameters (e.g., precursor concentration, linker‑to‑metal ratio, type of metal salt, and presence of water and/or modulators). However, the synthesis of new MOF gels remains rudimentary and largely empirical, due to insufficient accumulated knowledge to predict gel formation; thus, it must be optimized case by case.

Despite being complex and not yet fully understood, recent advances have shed light on the mechanisms of MOF gel formation.^[^ ^9^, ^10^, ^11^, ^12^ ^]^ Significant progress has been made in preparing various MOF gels, including UiO‐66,^[^ ^10^ ^]^ HKUST‐1,^[^ ^13^ ^]^ MIL‐53(Al),^[^ ^12^ ^]^ and ZIF‐8^[^ ^6^ ^]^ families.^[^ ^10^ ^]^ Yet a major bottleneck remains the scarcity of analytical techniques capable of providing detailed insight into gelation. Macroscopic characterization methods, such as powder X‐ray diffraction (PXRD), high‐resolution transmission electron microscopy (HRTEM), and dynamic light scattering (DLS), are typically employed, each with inherent limitations.^[^ ^4^ ^]^ PXRD data can be ambiguous, as the absence of sharp peaks may indicate either extremely small MOF nanocrystals or amorphous solids.^[^ ^10^, ^12^, ^13^ ^]^ HRTEM can, in principle, reveal crystalline nanoparticles invisible to diffraction, but detection is challenging because they are embedded in an amorphous gel matrix and are highly sensitive to electron beam damage, particularly at the ultrasmall nanoscale.^[^ ^10^, ^12^, ^13^ ^]^ Moreover, many MOFs, and especially at very small nanosizes, are extremely sensitive to the electron beam irradiation and their structures can be easily damaged during HRTEM imaging.^[^ ^14^ ^]^ DLS can monitor nanoparticle formation once they exceed ∼10 nm, providing some kinetic information.^[^ ^12^ ^]^ Overall, these macroscopic techniques require a minimum MOF nanoparticle size of at least a few nanometers. To access molecular‐level information, solid‐state nuclear magnetic resonance (ssNMR) spectroscopy has been employed. For example, ^27^Al experiments on Al–BDC gels revealed identical subunit structures and metal node environments throughout gelation.^[^ ^12^ ^]^ However, ssNMR requires specialized instrumentation, generally exhibits lower levels of automation than solution NMR, and demands significant expertise and long optimization times. Therefore, developing new analytical methods to probe early gelation at the molecular level is essential to correlate local structure with bulk properties and enable the rational design of next‑generation MOF gels.

A few years ago, we reported the first use of saturation transfer difference (STD) NMR to monitor the gelation of oxidized cellulose nanofibril dispersions upon heating, by following the STD signal of residually protonated water (HDO) in D_2_O gels, which reflects the population of confined or particle‐bound water.^[^ ^15^ ^]^ As a proof of concept, we employed a single saturation time (*t*_sat_) instead of the initial slope (STD_0_) obtained from the full STD build‐up curve. However, acquiring the full growth curve is preferred to minimize temperature‐dependent T_1_ effects and more accurately assess solvent–network interactions. To overcome the limitations of standard STD NMR, such as its dependence on gelator concentration, solvent accessibility, and network reorganization, in a subsequent publication we developed the spin diffusion transfer difference (SDTD) NMR protocol.^[^ ^16^ ^]^ This approach is independent of gelator and solvent concentrations, and allows to determine the spin diffusion rate at the particle‐solvent interface (D, in nm^2^ ms^−1^ units) and the minimum particle–solvent distance (r, in nm units) by normalizing the STD build‐up curve against the largest apparent STD factor and fitting it to the 1D diffusion Equation (1):^[^ ^16^ ^]^

(1) $$\mathrm{SDTD}\;=C\cdot \mathrm{erfc}\left[\frac{r}{2\cdot \sqrt{D\cdot t_{\mathrm{sat}}}}-b\right]$$

where the SDTD factor is the normalized intensity of the peak (STD(t)/STD(largest)), the independent variable (*t*_sat_^1/2^) is the square root of the saturation time (in units of ms^1/2^), *C* is the proportionally constant of the fit, erfc is the complementary error function, and b is a mathematical parameter to center the function around 0. The tangent of the curve mostly reflects changes in the diffusion rate *D* (larger slopes means larger *D*), whereas the lag phase is modulated by r (greater lag phase means longer r distances). The spin diffusion build–up curve (Equation 1) is simulated by varying either the spin diffusion rate *D* or the *r* distance while the other parameter is kept constant.

SDTD NMR enables in‐situ monitoring of solvent–gelator interactions during pre‐nucleation and gelation stages using standard solution state NMR. A faster SDTD curve growth (higher *D*) indicates increased fraction of bound‐solvent and/or greater solvent confinement/rigidification within the gel network. This allows accurate comparison of solvent structuration across different gel formulations and conditions, which is particularly valuable for time‐dependent systems like MOF‐gels.

From an application perspective, MOF gels and monoliths have been mainly explored for gas adsorption (e.g., hydrogen, methane,^[^ ^13^ ^]^ and toluene^[^ ^7^ ^]^), catalysis,^[^ ^17^, ^18^ ^]^ and environmental decontamination.^[^ ^19^ ^]^ Their potential for biomedical applications lies in their hierarchical micro‐ and mesoporous structures, which enable the co‐encapsulation of drugs with diverse sizes and chemical properties, combined with tunable release kinetics, stimulus‐responsiveness, and the intrinsic biocompatibility of certain MOFs. Reported drug delivery examples rely exclusively on hybrid MOF gels (MOFs embedded in polymer matrices such as cellulose, polyurethane, or PLGA),^[^ ^20^, ^21^, ^22^ ^]^ using small drugs (e.g., 5‐fluorouracil,^[^ ^20^ ^]^ brimonidine,^[^ ^21^ ^]^ doxorubicin, or celecoxib^[^ ^22^ ^]^) pre‐loaded into the MOF particles and further incorporated into the polymer matrix, thus neglecting the meso‐/macropores of pristine MOF gels. On the other hand, encapsulating larger biomolecules (e.g., carbohydrates or proteins) requires in‐situ incorporation during gel formation under mild conditions to preserve their structural integrity. To date, only one study has reported the inclusion of glycosaminoglycans (heparin, hyaluronic acid, chondroitin sulfate, and dermatan sulfate) in the precursor mixture for MAF‐7, which in some cases yielded a gel‐like material.^[^ ^23^ ^]^ It seems therefore clear that the field of MOF gels for biomedical purposes is still in its infancy, requiring further research to control biomolecule encapsulation and elucidate its effects on gel properties.

In this work, we focus on UiO‑66 gels [([Zr_6_O_4_(OH)_4_(BCD)_6_], UiO = Universitetet i Oslo; BCD = 1,4‐benzenedicarboxylate] due to their biocompatibility, low cytotoxicity, high drug‑loading capacity,^[^ ^24^ ^]^ and suitability as a model for other Zr‑MOF gels.^[^ ^10^, ^17^, ^25^ ^]^ We first establish an optimized low‑temperature, acid‑free synthesis that enables the in‐situ encapsulation of biomolecules sensitive to harsh conditions. As a proof of concept, we encapsulate a therapeutic glycolipid within the MOF gel. We demonstrate that the combined analysis of STD NMR and SDTD NMR growth curves of water over time allows to follow, in real time, the population of network‐bound water and water rigidification around the metal–organic clusters under different conditions (with and without acid, and during in‐situ biomolecule encapsulation). This solution state NMR approach thus provides molecular‐level insights into the early nucleation and gelation stages of UiO gels. Finally, we correlate the solvent structuration at the molecular level, as characterized by NMR, with the macroscopic properties of the resulting gels.

## Results and Discussion

### Preparation of UiO‐66 MOF Gels

**Scheme 1:**
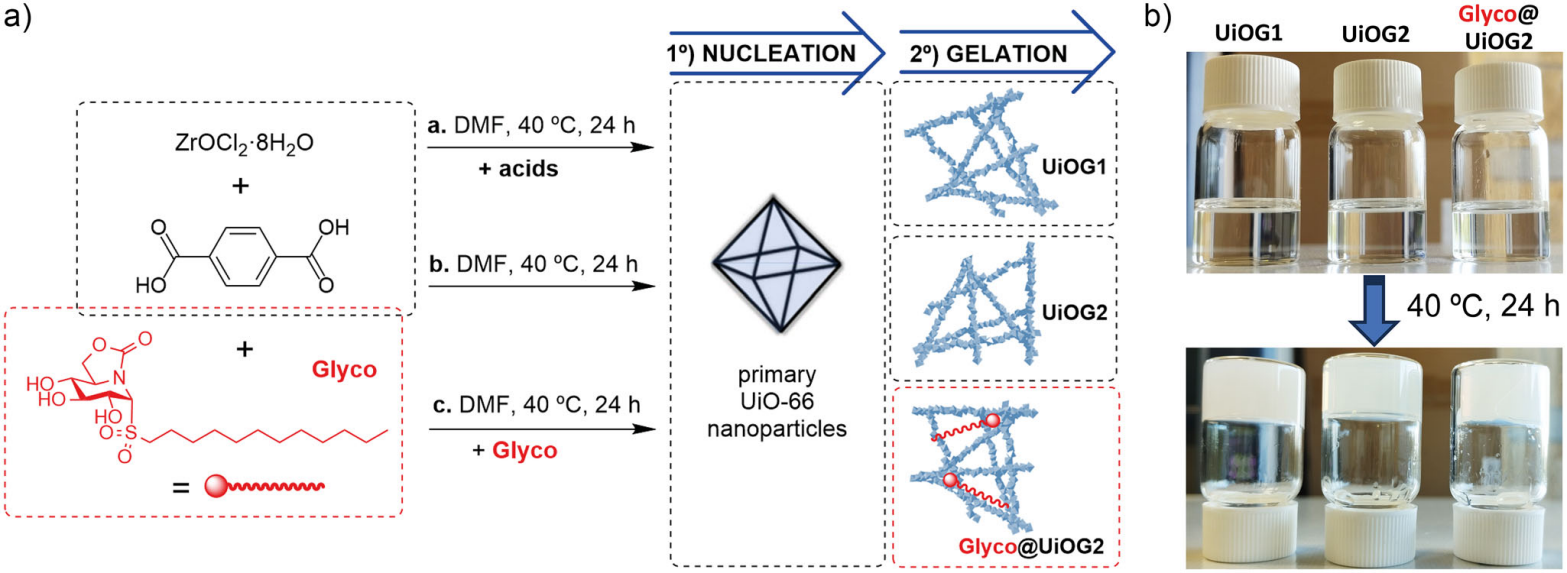
a) Schematic representation of the preparation of UiO‐66‐based gels (UiOG), including the in‐situ incorporation of a glycolipid (Glyco) compound within the gel matrix. b) Photographs of the precursors mixture before and after the gels formation.

Inspired by the method described by Bennett et al.,^[^ ^10^ ^]^ we prepared UiO‐66 gels (i.e., [Zr_6_O_4_(OH)_4_(BDC)_6_]) using a modified protocol to achieve the gelation at 40 °C, much lower than the 100 °C previously reported for Zr‐based MOF gels.^[^ ^10^, ^26^, ^27^ ^]^ Following earlier reports, we selected ZrOCl_2_·8H_2_O as the metal source, as its water content is sufficient to form the initial [Zr_6_O_4_(OH)_4_]^12+^ clusters. However, under the reported conditions, gelation did not occur at 40 °C, likely because nucleation was inhibited at such a low temperature. It is worth noting that Zr‑MOF gel formation involves two steps: i) nucleation of primary UiO‑66 nanoparticles and ii) their aggregation via van der Waals interactions to form a gel. We found that high precursor supersaturation enabled nucleation at 40 °C. In practice, increasing the concentrations of ZrOCl_2_·8H_2_O and H_2_BDC, while keeping the linker‐to‐metal ratio constant, was critical for low‑temperature gelation. Under optimized conditions (see ESI for details, Table S1), we obtained non‑flowing gels at 40 °C either with or without acetic acid, named UiOG1 and UiOG2, respectively (Scheme 1). We also explored the in‐situ incorporation of a therapeutic glycolipid (Glyco, Scheme 1a) with anti‐inflammatory activity,^[^ ^28^ ^]^ (1*R*)‐1‐dodecylsulfonyl‐5 *N*,6*O*‐oxomethylidenenojirimycin, during UiOG2 formation. Glyco was added to the precursor mixture (1.2 mmol H_2_BDC, 0.8 mmol ZrOCl_2_·8H_2_O) at two concentrations (0.02 and 0.08 mmol; Glyco‐to‐Zr molar ratios 0.025 and 0.1).

Since Glyco did not inhibit gelation, we proceeded with the higher concentration, naming the resulting gel Glyco@UiOG2. Visual inspection during incubation confirmed that lower temperatures slowed gel formation; at 40 °C, the non‐flowing state was reached in 18–20 h, as evaluated by the tube inversion test (Scheme 1b). Notably, Glyco@UiOG2 appeared more opaque and detached from the glass vial wall, likely as a consequence of the glycolipid's amphiphilic nature. Although the use of acidic modulators showed no apparent effect on gel formation by visual inspection (UiOG1 vs UiOG2), we further examined its influence on bulk properties and molecular‐level gelation. All gels were isolated by centrifugation as wet, non‐flowing solids, solvent‐exchanged with *N,N*‐dimethylformamide (DMF) and ethanol (EtOH), and freeze‐dried to obtain powders suitable for further XRD, FTIR and TGA analysis. Lyophilization was preferred over conventional drying at 200 °C, typically used to obtain monolithic xerogels,^[^ ^10^ ^]^ in order to preserve the gel macrostructure and prevent biomolecule degradation.

### Macroscopic Characterization of MOF Gels

**Figure 1:**
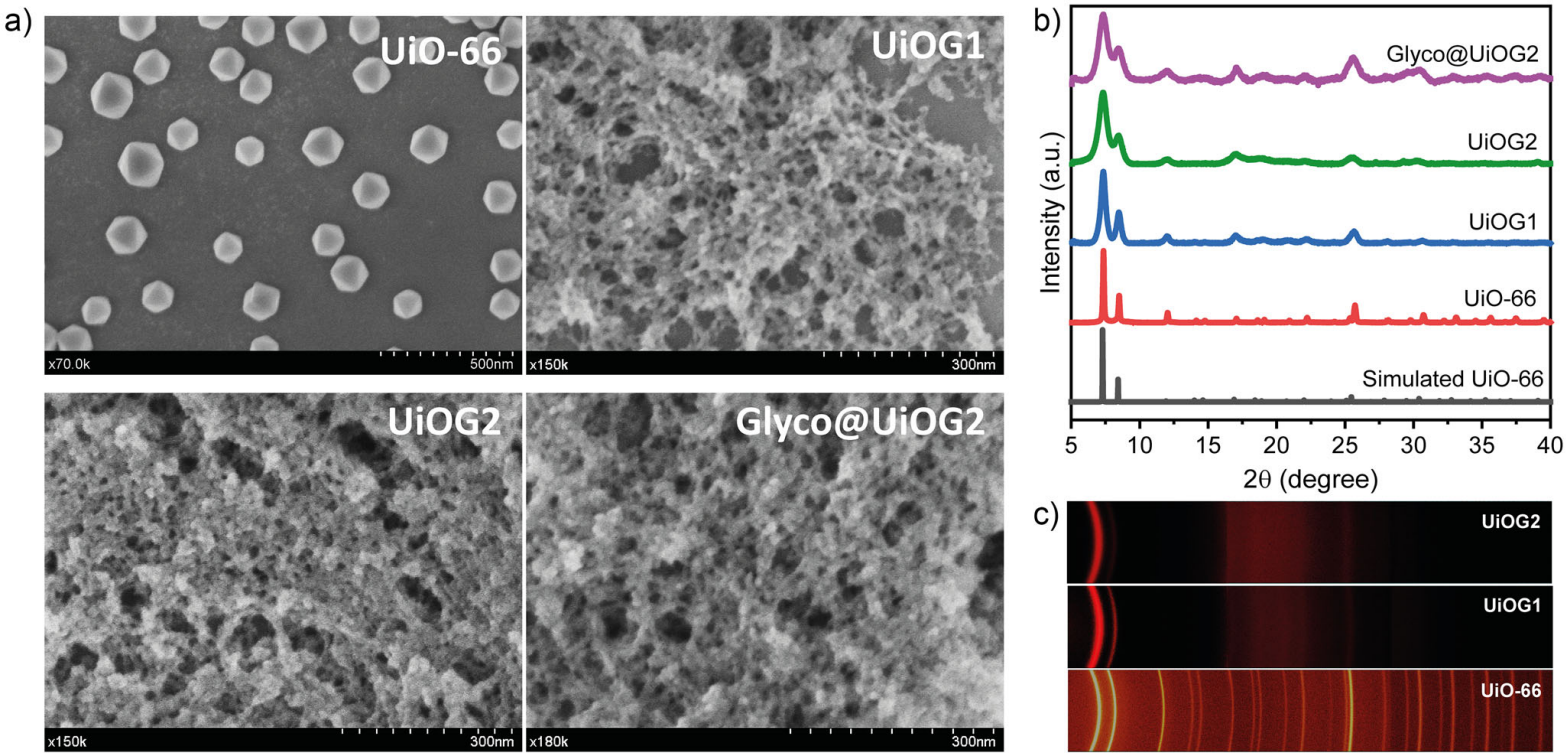
a) SEM images of the as prepared gels and microcrystalline UiO‐66 nanoparticles as a reference. b) XRD patterns of the gels after ethanol‐exchange. For comparison, the simulated UiO‐66 pattern and that of UiO‐66 nanoparticles are also shown. c) 2D‐μ‐XRD patterns in the 2θ range of 5°–40° for UiOG1 and UiOG2 gels, compared with the powdered UiO‐66 nanoparticles.

The macroscopic and structural characterization of the UiO‐66‐based gels revealed a series of features that clearly distinguish these gel‐materials from its microcrystalline counterpart. Scanning electron microscopy (SEM) revealed that all gels exhibit a sponge‐like architecture composed of densely packed UiO‐66 nanoparticles interconnected by mesoporous voids throughout the network (Figure 1a). Such a hierarchical organization is characteristic of MOF gels and is crucial for their appealing properties, as the interparticle mesopores facilitate solvent diffusion and provide accessible sites for guest molecules. Notably, the SEM images indicate a homogeneous texture without any evidence of phase separation or morphological heterogeneity. The homogeneous distribution of Zr throughout the gels was confirmed by EDX mapping (Figure S1).

To further probe the crystalline structure, we performed X‐ray diffraction (XRD) on the gels (UiOG1, UiOG2, and Glyco@UiOG2). Remarkably, all three samples exhibited diffraction patterns that could be indexed to the fcu topology of UiO‐66 and matched both the microcrystalline UiO‐66 reference (∼100 nm particle size) and the simulated pattern (Figure 1b). Nevertheless, the XRD patterns of the gels were characterized by broad and less intense peaks, particularly for the reflections centered at ca. 7° and 8.5° 2θ, compared to the sharp peaks of the microcrystalline control. This pronounced broadening indicates a significant reduction in coherent domain size, consistent with the formation of ultrasmall UiO‐66 nanoparticles and the loss of extended long‐range order within the gel network. While the broadening primarily reflects smaller coherent domains, a contribution from structural defectivity (e.g., mosaic spread or missing‐linker vacancies) is also expected and likely coexists, consistent with the slightly increased linker deficiency derived from thermogravimetric analysis, as discussed later. Moreover, two‐dimensional μ‐diffraction (2D‐μ‐XRD) mapping was performed at different regions across the gels to assess their structural homogeneity (Figures 1c and S2). The diffraction patterns displayed two broad yet well‐defined Debye rings with uniform intensity, confirming the homogeneous nanocrystalline nature of the gels. The crystallinity index values obtained from the 2D‐μ‐XRD maps remained nearly constant across all regions, demonstrating a uniform amorphous‐to‐crystalline ratio throughout the samples (Table S2).

From the Scherrer analysis of the XRD peaks, we estimated average crystalline domain sizes of 20.6 nm for UiOG1 and 12.6 nm for UiOG2, which is substantially smaller than the 78 nm observed for the microcrystalline reference. Glyco@UiOG2 showed a domain size of 12.0 nm, virtually identical to UiOG2, suggesting that glycolipid encapsulation does not hinder nucleation nor further limit crystal growth. Interestingly, the slightly smaller crystalline domains of UiOG2 (prepared in the absence of acetic acid) compared to UiOG1 (with acetic acid) align with the known role of acetic acid as a modulator that typically favors larger crystal growth in UiO‐66 syntheses.^[^ ^29^ ^]^ This behavior highlights the delicate interplay between nucleation kinetics, modulator presence, and the formation of extended gel networks.

**Figure 2:**
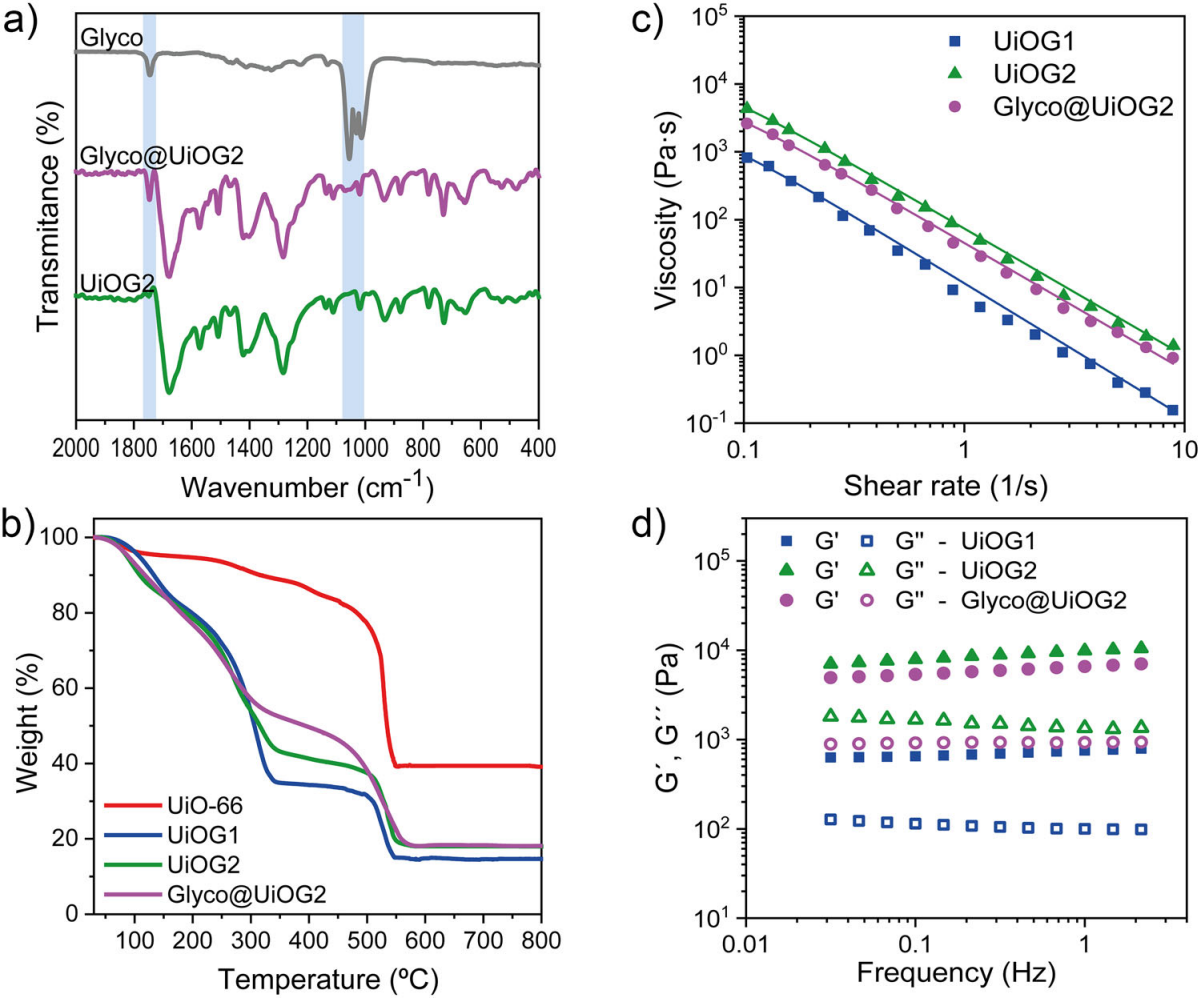
a) FT‑IR spectra of the UiOG2 gel, the glycolipid (Glyco), and Glyco@UiOG2. Blue areas highlight the characteristic peaks of the glycolipid. b) TGA curves of the gels after ethanol exchange and freeze‑drying. c) Flow curves (viscosity as a function of shear rate) of the as‑prepared gels. Lines represent fits to the experimental data using the Cross model, with parameters provided in Table S3. d) Frequency sweep tests (G′ and G′’ vs frequency) at 25 °C for the different gels.

The successful encapsulation of the glycolipid within the MOF structure was unambiguously confirmed by Fourier‐transform infrared spectroscopy (FT‐IR). The spectrum of Glyco@UiOG2 displayed new, intense bands not present in the parent gel (UiOG2) (Figures 2a and S3). Specifically, a strong carbonyl stretching band at 1744 cm^−1^ and characteristic vibrations in the 1250–1000 cm^−1^ region, attributable to S═O and C–O modes, confirmed the molecular fingerprint of the glycolipid. These features indicate that the guest molecules are effectively retained within the gel network after washing and drying, which is essential for subsequent functional applications. Moreover, the FTIR spectra of the gels display the characteristic UiO‐66 vibrational bands without additional broad features typical of amorphous zirconia phases, confirming the structurally homogeneous nature of the gels.

Thermal gravimetric analysis (TGA) further elucidated the composition, solvent content, and framework thermal stability of the gels, in comparison with microcrystalline UiO‑66 particles (Figures 2b and S4). Pristine UiO‑66 nanoparticles displayed the typical profile with a rapid weight loss at about 500 °C, which corresponds to the framework decomposition. In contrast, the gels exhibited a more complex, multistep decomposition profile, revealing their distinct structural and compositional nature. The first weight‑loss stage, occurring between 100 °C and 270 °C, is assigned to the release of strongly coordinated solvents, primarily water and ethanol, trapped in the extensive mesoporous network and at defect sites. The second stage, spanning 270 °C–500 °C, corresponds to the gradual elimination of residual DMF molecules and partial node dehydration of the Zr_6_‑oxo clusters, a process whose extent and temperature range are slightly shifted in the gels, as typically observed in defective UiO‐66 frameworks with undercoordinated sites. The final stage, around 500 °C, marks the complete framework decomposition as the organic linkers combust. It is important to note the larger fraction of coordinated solvent molecules found in UiOG2 compared to UiOG1. Glyco@UiOG2 exhibited a distinctive thermal profile, with a more gradual weight loss within the 400 °C–550 °C range. This broadened decomposition interval resembles that observed in glycolipid‐loaded ZIF‐8 nanostructures^[^ ^30^ ^]^ and is indicative of an intimate guest–host interaction that retards thermal degradation. Analysis of the TGA curves also allowed quantification of framework defectivity and glycolipid content; see ESI for calculations (Table S3). The fraction of missing linkers was higher in the gels (6.5% for UiOG1 and 5.4% for UiOG2) than in the microcrystalline UiO‐66 (2.8%), which is fully consistent with the literature on MOF gels, where rapid nucleation and aggregation into a network typically introduce structural vacancies. Finally, the glycolipid content of Glyco@UiOG2 was estimated from TGA by assuming a constant Zr/linker ratio. A loading of 12.4 wt% was calculated, which is in excellent agreement with the theoretical value of 13.5 wt% based on the initial feed. This consistency between experimental and theoretical loadings not only validates the encapsulation process but also demonstrates that the gel matrix can accommodate substantial amounts of hydrophobic guest molecules without compromising its crystalline integrity.

To assess the porosity of the gels, lyophilized samples were analyzed by N_2_ adsorption–desorption at 77 K (Figures S5 and Table S4). Both gels exhibited type IV isotherms with hysteresis loops, typical of mesoporous materials. Their N_2_ adsorption capacity was markedly lower than that of microcrystalline UiO‐66, reflecting their less compact structure and smaller crystalline domains. UiOG2 had a slightly higher surface area (S_BET_ = 585 m^2^ g^−1^) and pore volume (*V*_total_ = 0.35 cm^3^ g^−1^) than UiOG1 (502 m^2^ g^−1^, 0.28 cm^3^ g^−1^), indicating a more open network. Both gels showed enhanced external surface area and mesoporosity relative to UiO‐66 (Table S4), consistent with the hierarchical assembly of nanosized UiO‐66 units into a continuous gel structure.

Rheological characterization of the as‐prepared gels (UiOG1, UiOG2, and Glyco@UiOG2) provided further insights into their internal network organization. It is worth noting that rheological measurements were performed on the gels in their native wet state, which represents the relevant condition for biomedical applications. All samples exhibited pronounced shear‐thinning and thixotropic behavior (Figure 2c), typical of pseudoplastic gels that flow under stress manual stirring for 30 s, Figure S6) and recover their solid‐like state after resting. Fitting of the flow curves with the Cross model (*R* ^2^ > 0.99; Table S5) yielded a much higher zero‐shear viscosity for UiOG2 (*η*_0_  =  23 823 Pa·s) than for UiOG1 (*η*_0_  =  4 341 Pa·s), reflecting a more cohesive and interconnected internal network in UiOG2. This correlates with the higher solvent coordination detected by TGA and the smaller crystalline domains observed by XRD. Upon glycolipid incorporation, *η*_0_ decreased to 13 976 Pa·s, indicating a slight network disruption while preserving the characteristic shear‐thinning behavior.

Frequency‐sweep experiments (Figure 2d) confirmed their gel‐like nature, with the elastic/storage modulus (G′) consistently exceeding the loss modulus (G′’) across the entire frequency range. UiOG2 displayed the highest storage modulus (*G*′ = 10 460 Pa ≈ 10 kPa), whereas UiOG1 was softer (*G*′ = 790 Pa ≈ 0.8 kPa). These values for our wet gels are several orders of magnitude lower than those reported for solvent‐free UiO‐66 monoliths or xerogels (E ≈ 10 GPa),^[^ ^10^ ^]^ reflecting the solvent‐rich, dynamically reversible nature of the hydrated gels, which is precisely the desirable behavior for soft and injectable materials intended for biomedical applications. Indeed, our gels exhibit G′ and G′’ values very similar to those reported for ZIF‐8‐based supramolecular MOF gels, which display mechano‐responsive elasticity under cyclic strain.^[^ ^31^ ^]^ Overall, UiOG2 forms the most cohesive and reversible network, while glycolipid incorporation slightly softens the matrix without compromising the pseudoplastic behavior essential for processability and biomedical use.

### In‐Situ Monitoring of Solvent Structuration During UiO‑66 Gel Formation by STD and SDTD NMR Analysis

**Figure 3:**
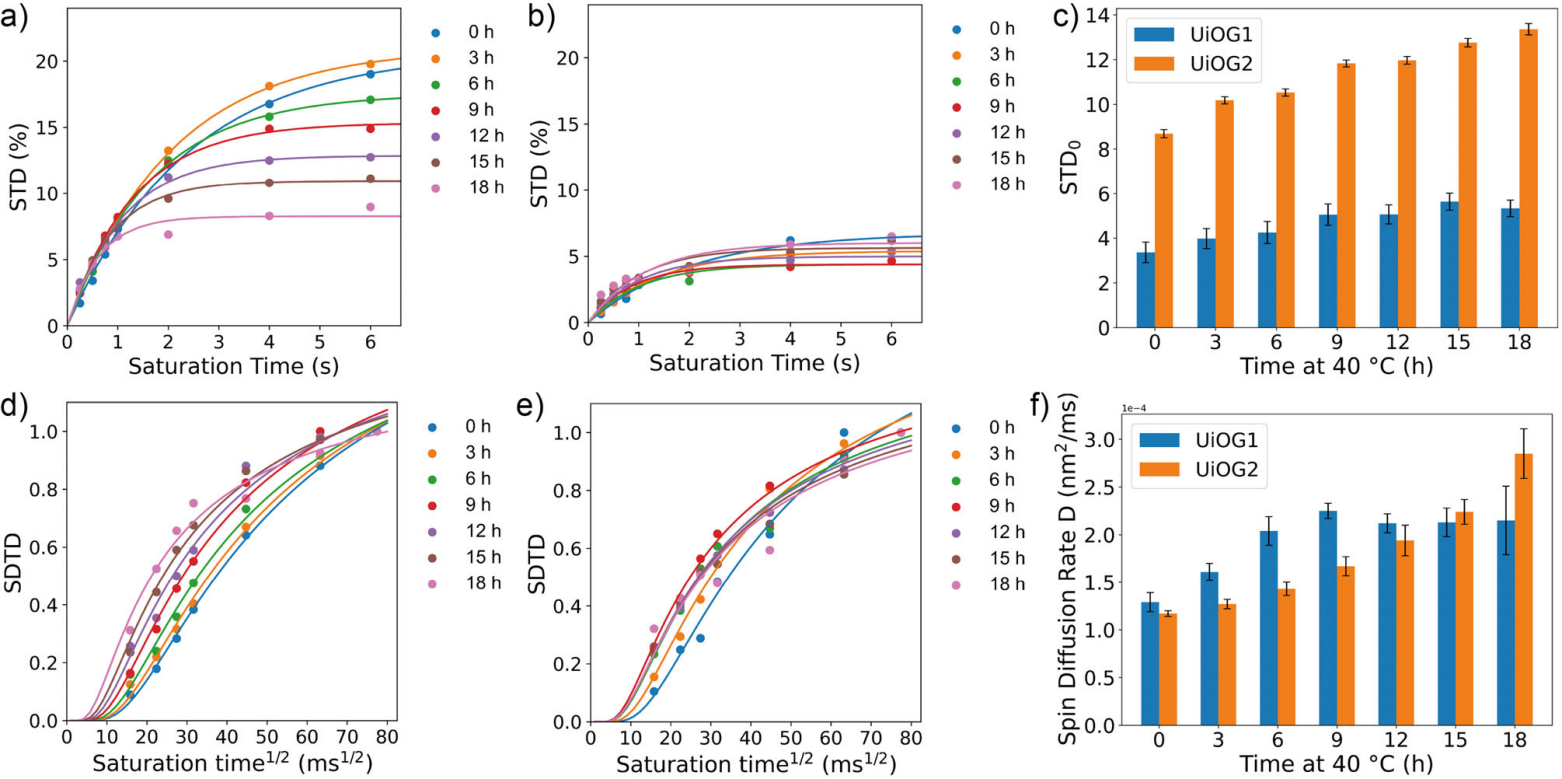
a,b) STD build‐up curves for UiOG2 (a) and UiOG1 (b) gels recorded during the in‐situ gelation (40 °C, 18 h, 600 MHz). c) Evolution of the STD_0_ of water, determined from STD build‐up curves collected during in‐situ gelation of UiOG1 (blue bars) and UiOG2 (orange bars). Error bars are expressed as relative errors. d,e) SDTD build‐up curves for UiOG2 (d) and UiOG1 (e) gels recorded during the in‐situ gelation (40 °C, 18 h, 600 MHz). f) Evolution of the spin‐diffusion rate (D) at the UiO–water interface, extracted from SDTD fits at different time intervals during in‐situ gelation of UiOG1 (blue bars) and UiOG2 (orange bars). Error bars are expressed as the standard deviation associated with the fitted values.

Aiming to better understand UiO‐66 gel formation at the molecular level and to rationalize the macroscopic differences observed among UiOG1, UiOG2, and Glyco@UiOG2, we carried out in‐situ NMR studies of UiO–water interactions during the formation of UiOG1 and UiOG2 gels over an 18‐h period, starting from the moment the air flow surrounding the NMR tube reached 40 °C. In particular, STD NMR experiments were acquired at different saturation times at seven different time intervals to construct the STD build‐up curves (Figure 3a,b). From these, we determined the STD initial slopes (STD_0_; Figure 3c) and built the SDTD growth curves (Figure 3d,e). Fitting of the latter to Equation (1) allowed to obtain the spin diffusion rate at the UiO‐water interface (D; Figure 3f).

The STD build‑up curves of UiOG2‐water interactions revealed a clear temporal evolution, with maximum STD intensities of about 20% at the beginning decreasing to about 7% after 18 h (Figure 3a), and STD_0_ values showing a progressive increase over time beyond the associated error (Figure 3c, orange bars). In contrast, UiOG1 exhibited much lower STD responses, with maximum STD intensities of only ∼5% (Figure 3b), and STD_0_ values not showing a consistent trend throughout the seven time points that were monitored (Figure 3c, blue bars). Visual examination of the SDTD build‐up curves reflected the same behavior, i.e., UiOG2 displayed a progressively faster SDTD growth with time (the speed of SDTD growth is directly proportional to the spin diffusion rate D), qualitatively reflecting the continuous increase of water confinement during gel formation. Time evolution analysis of D further confirmed the increase of the spin‑diffusion rate beyond the error (Figure 3f, orange bars) and, thus, the significant rise of water structuration as the gel is formed. UiOG1, on the other hand, showed a less defined evolution (Figure 3f, blue bars). These results provide the first experimental evidence of optimal conditions enabling the slow and progressive formation of UiO‑66 gels (i.e., conditions of UiOG2), where gradual assembly allows for detailed monitoring of solvent structuration at the molecular level, a key factor for the rational design of new MOF‐gels. Conversely, gel formation under UiOG1 conditions was more difficult to monitor, likely due to the presence of acetic acid, which initially caps the Zr nodes and slows down linker incorporation. Recent studies on microcrystalline UiO‑66 have proposed that nucleation does not start with the isolated Zr‐node, but with the formation of a small MOF oligomer (germ), stabilized by a kinetic network effect.^[^ ^32^ ^]^ In the presence of excess acetate, linker incorporation is initially inhibited, and the rate‑limiting step becomes the dissociation of the modulator from the node. Once a germ forms, linker substitution accelerates and the framework grows rapidly. This acid‑assisted dissociative mechanism leads to faster crystal growth after nucleation, which could explain the larger crystalline domains observed for UiOG1 (20.6 nm) compared to UiOG2 (12.6 nm). While this process has been recently described for the formation of microcrystalline UiO‑66 particles,^[^ ^32^ ^]^ our results suggest that a similar mechanism may also operate in UiO‑66 gels, providing a molecular explanation for the less gradual evolution of UiOG1 observed in our NMR studies.

The analysis of STD_0_ over time indicates a higher fraction of UiO‑bound water during gel formation in the absence of acid (UiOG2), as reflected by the much greater STD_0_ values compared to UiOG1 (Figure 3c). This agrees with TGA, which shows a larger fraction of coordinated solvent molecules in UiOG2, and with XRD data revealing its slightly smaller mean particle size (i.e., higher surface area). In contrast to STD_0_, D values were initially greater for UiOG1 up to 9 h, then displaying similar values (within the error) up to 18 h (gel state). UiOG2, instead, showed a progressive increase of D and a higher spin‑diffusion rate at the last time point (18 h; Figure 3f). As STD_0_ mainly reflects the fraction of bound water and D is sensitive to the extent of solvent structuration/rigidification within the gel network, the similar time‑dependent behavior in UiOG2 suggests that both a larger fraction of network‑bound water and greater water confinement/rigidification contribute to gel formation. The evolution of D in UiOG1 is consistent with a faster prenucleation of clusters that leads to early water rigidification, whereas UiOG2 evolves more gradually but reaches higher solvent structuration in the gel state. Accordingly, UiOG2 ultimately presents a larger fraction of both network‑bound and rigidified water, which is consistent with the macroscopic observations, particularly TGA and rheology, and correlates with its higher viscosity (Figure 2c) and storage modulus (*G*’; Figure 2d). Overall, this supports a more interconnected network of smaller crystalline particles holding a greater fraction of structured solvent molecules, which explains the more viscous gel‑like behavior of UiOG2.

The in‐situ formation of Glyco@UiOG2 showed similar trend for D, progressively increasing over a 12 h timeframe, and with values similar to UiOG2; this suggests a quite similar degree of water structuration in both gels. STD_0_, however, did not change significantly over the experimental time, and displayed much lower values than in UiOG2 (Figures S7 and S8). This indicates a lower population of UiO‐bound water in the presence of the glycolipid, as expected due to its hydrophobic character, which correlates with the slightly reduced viscosity and elasticity (*G*’) of Glyco@UiOG2 (Figure 2c,d).

Similar to the characterization of water‐UiO interactions, we attempted a similar analysis for the interactions with DMF, i.e., the major solvent present in the UiO samples. While DMF‐UiO interactions were observed, the STD response of the DMF peak was very weak at long saturation times (1%–2% at 4‐to‐6 s *t*_sat_), and at the noise level for shorter times. This is due to DMF being in large excess, so that the fraction of bound DMF is very small and, hence, the STD intensity becomes compromised by the detection limit of the technique.

It is also noticeable that the ^1^H NMR signal of water showed a shift either toward high (shielding) or low (deshielding) field in the presence or absence of acid during gel formation (UiOG2 or UiOG1, respectively; Figures S9–S13). This behavior can be attributed to the protonation state of the carboxylate groups of the terephthalate (BDC) linkers upon acid addition, which alters the local chemical environment of water molecules either coordinated to the Zr_6_ nodes or structured water within the gel matrix. As the acidity of the medium increases, protonation reduces the electron density around the linker coordination sites, potentially weakening water–framework interactions. This leads to changes in the magnetic field experienced by water protons, resulting in a downfield shift of the NMR peak. Similar effects have been reported in previous studies using ^2^H NMR to monitor UiO‐66 formation in the presence of an excess of acetic acid as modulator, highlighting the sensitivity of solvent signals to the protonation state of the framework.^[^ ^32^ ^]^

## Conclusions

Our study reports the preparation of UiO‑66‑type MOF gels under mild conditions (40 °C, in the absence of acidic modulators) that provide a biocompatible environment suitable for the in‐situ encapsulation of sensitive biomolecules. This approach allows their efficient incorporation into the MOF network under non‐degrading conditions. We show that, despite the low water content in our UiO gels, water can be effectively used as a reporter of the gelation process by monitoring its interactions with the MOF network. This finding further highlights the crucial role of using an octahydrated Zr salt as precursor for UiO gel formation.

Further, we demonstrate that the combined analysis of STD initial slopes and spin‑diffusion rates from SDTD build‐up curves during in‐situ gel formation provides a powerful tool to monitor the evolution of the fraction of bound solvent and the degree of solvent structuration from the early prenucleation stages to the final gel state. In addition, we show that this progressive real time monitoring is favored in the absence of acid as it slows down the gelation process.

Importantly, our NMR data are in excellent agreement with macroscopic characterization of the gels, i.e., the higher fraction and/or rigidification of bound water in UiOG2, followed by Glyco@UiOG2 and UiOG1, correlate with the larger amount of coordinated solvent molecules determined by TGA, the smaller crystalline domains estimated from XRD, and the differences in viscosity and elasticity revealed by rheology. We also show that the encapsulation of a biomolecule (specifically a therapeutic glycolipid) within UiO‐gels, while modifying the macroscopic properties to a moderate extent, does not compromise the integrity of the gel network.

Overall, our work establishes a multiscale characterization approach that bridges molecular‑level solvent–MOF interactions with macroscopic gel properties, providing mechanistic insight into MOF gel formation. We believe that these findings pave the way for the rational design of novel UiO‑based gels optimized for the in‐situ encapsulation of sensitive biomolecules, while ensuring robust gel formation and maintaining the essential functional properties of MOF gels.

## Supporting Information

The Supporting Information file includes full experimental procedures, characterization details, and additional supporting data and figures. The authors have cited additional references within the Supporting Information (Refs. [33 and 34]).

## Author Contributions

**J.C.M.‐G**.: Methodology, investigation, formal analysis, validation, writing—original draft, writing—review and editing; **F.G.M**.: Investigation, writing—review and editing; **E.M.S.‐F**.: Investigation, funding acquisition, writing—review and editing; **J.S**.: Investigation, writing—review and editing; **J.A**.: Funding acquisition, writing—review and editing; **C.C.‐C**.: Conceptualization, methodology, investigation, formal analysis, validation, supervision, funding acquisition, writing—original draft, writing—review and editing. All authors have reviewed the manuscript before submission.

## Conflict of Interests

The authors declare no conflict of interest.

## Supporting information



Supporting Information

## Data Availability

The data that support the findings of this study are available in the Supporting Information of this article.
