# Supplementary material for: Insights into the Controlled Formation of Zr‐Based Metal–Organic Gels: Linking Macroscopic Properties with Molecular Information from Solution State NMR
Source: Angew Chem Int Ed Engl. 2026 Jan 19;65(9):e20987. doi: 10.1002/anie.202520987 (PMC12930015; doi:10.1002/anie.202520987)
Supplement: Supplementary file 1 — Supporting Information [file ANIE-65-e20987-s001.pdf]

SUPPORTING INFORMATION

Insights into the Controlled Formation of Zr-based Metal-Organic Gels: Linking Macroscopic Properties with Molecular Information from Solution State NMR

Juan C. Muñoz-García,[a]* Francisco G. Moscoso,[b] Elena M. Sánchez-Fernández,[c] Jenifer Santos,[d] Jesús Angulo,[a] Carolina Carrillo-Carrión[a]*

[a] J. C. Muñoz-García, J. Angulo, C. Carrillo-Carrión
Institute for Chemical Research (IIQ)
CSIC – University of Seville
41092 Seville, Spain
E-mail: juan.munioz@iiq.csic.es, carolina.carrillo@csic.es

[b] Center for Nanoscience and Sustainable Technologies (CNATS)
Department of Physical, Chemical and Natural Systems
Pablo de Olavide University
41013 Seville, Spain

[c] Department of Organic Chemistry
Faculty of Chemistry, University of Seville
41012 Seville, Spain

[d] Department of Health and Biomedical Sciences,
Faculty of Health Sciences, Universidad Loyola Andalucía
41704 Dos Hermanas, Seville, Spain

TABLE OF CONTENTS	Page
**S1. Chemicals and Instrumentation**	2
**S2. Synthesis and purification of gels**	3
**S3. Additional structural characterization of gels**	4
**S4. Additional NMR data of gel formation process**	9
**S5. References**	12

S1. Chemicals and Instrumentation

Chemicals: All reagents were obtained from commercial sources and were used without further purification. These reagents were 1,4-benzenedicarboxylic acid (H_2BDC; Sigma-Aldrich, 98%); zirconyl chloride octahydrate ($ZrOCl_2·8H_2O$; Sigma-Aldrich, 98%); glacial acetic acid (HAc; Sigma-Aldrich, 100%); N,N-dimethylformamide anhydrous (DMF; Sigma-Aldrich, 99.8%), ethanol (EtOH; Sigma-Aldrich, 99.5%), deuterated methanol (methanol-d4; Sigma-Aldrich, ≥99.8 atom % D) and deuterated dimethylformamide (N,N-dimethylformamide-d7; Sigma-Aldrich, ≥99.5 atom % D). The glycolipid, (1*R*)-1-dodecylsulfonyl-5*N*,6*O*-oxomethylidenenojirimycin, was prepared according to a previously reported procedure [1].

Scanning Electron Microscopy (SEM): SEM images were acquired with a HITACHI S4800 field emission microscope operating at 2 kV in secondary electron and backscattered electron modes. Samples were prepared by drying a diluted dispersion of the gels on a silicon wafer substrate. Elemental composition mapping of the gels was performed by Energy Dispersive X-ray analysis (EDX) using the same SEM instrument coupled to a Bruker-X Flash-4010 EDX detector and operating at 5 kV.

X-Ray Diffraction (XRD): X-ray analysis of the gels was performed using a Bruker D8-Advance Diffractometer operated at 50 kV and 1 mA for Cu kα (λ=1.5418 Å). All measurements were collected in 2D mode using a Bruker EIGER-2R 500K Detector (θ =11.9º, γ = 6º), with a step of 11.7º per 1800 s in the range 5º-40º (2θ). The detector distance from the samples was fixed at 35 cm. The gel samples were deposited in a zero-background Si holder (KS Analytics) for the measurements.

Fourier-transform infrared spectroscopy (FTIR): FTIR spectra were acquired using an FT-IR-4700 (Jasco) with a scanner velocity of 2.2 kHz and a resolution of 4 cm^{-1} in the range 400-4000 cm^{-1}. The gel samples were deposited on a silicon wafer substrate to obtain the transmittance infrared.

Thermogravimetric analysis (TGA): TGA was performed by analyzing approximately 40 mg of the lyophilized gel samples under an air atmosphere using a TGA PT-1000 (Linseis) instrument with a heating profile from 30 to 800 ºC and a heating rate of 10 ºC/min.

N_2 physisorption analysis: N_2 sorption isotherms at 77 K of the gel samples were carried out in a Micromeritics Tristar II 3020 system. Before analysis, samples were degassed under vacuum for 18 h at 120 °C. The apparent surface areas were calculated from the Barrett–Emmett–Teller (BET) method in the pressure interval P/Po = 0.01 - 0.3 (being Po the saturation pressure). Pore volume and external surface area were calculated by the t-plot method. S_{BET} refers to the Brunauer–Emmett–Teller surface area; S_{micro} to the micropore surface area; S_{ext} to the external surface area; V_{micro} to the micropore volume; V_{meso} to the mesopore volume; and V_{total} to the total pore volume.

Rheological characterization: The rheological properties of the samples were measured using a rotational rheometer model Haake Mars 40 (Thermo Fisher Scientific, Karlsruhe, Germany) equipped with a serrated parallel plate-plate geometry (diameter 35 mm, gap 1 mm). All experiments were conducted at a controlled temperature of 25 °C. Flow behavior was assessed by recording shear stress over a range of shear rates from 0.1 to 10 s^{-1}. Oscillatory tests were performed to evaluate the storage modulus (G') and loss modulus (G"), with frequency sweeps conducted from 3 to 0.03 Hz. Prior to these tests, stress sweep measurements at 1 Hz were used to define the linear viscoelastic region (LVR).

Nuclear Magnetic Resonance Spectroscopy (NMR): Solution state NMR experiments were performed on a Bruker Avance III spectrometer equipped with a 5 mm QCI cryoprobe operating at a frequency of 600.21 MHz (^1H). Saturation transfer difference (STD) NMR experiments were acquired at 313 K during in situ formation of MOF gels using a train of 50 ms Gaussian shaped pulses for selective

saturation of the MOF particles, using an *on*– and *off-resonance* frequency of −1 and 40 ppm, respectively. Saturation times ranging from 0.25 to 5 s were employed. A constant time length per scan (saturation time + recycle delay) of 6.1 s was used. STD NMR experiments were performed with 128 scans or less (with a minimum of 32 scans), in inverse relation to the saturation time, and 8 dummy scans.

S2. Synthesis of gels and purification

UiOG1 synthesis. The gel synthesis was performed in a 12 mL glass vial by dissolving 1.2 mmol (0.2 g) H_2BDC and 0.8 mmol (0.26 g) $ZrOCl_2{\cdot}8H_2O$ in 3.5 mL (45 mmol) DMF, followed by the addition of 0.16 mL (2.8 mmol) HAc. The precursors mixture was incubated in an oven at 40 °C for 24 h.

UiOG2 synthesis. The gel synthesis was performed in a 12 mL glass vial by dissolving 1.2 mmol (0.2 g) H_2BDC and 0.8 mmol (0.26 g) $ZrOCl_2{\cdot}8H_2O$ in 3.5 mL (45 mmol) DMF, and the resulting mixture was incubated in an oven at 40 °C for 24 h.

Glyco@UiOG2 synthesis. The gel synthesis was performed in a 12 mL glass vial by dissolving 1.2 mmol (0.2 g) H_2BDC and 0.8 mmol (0.26 g) $ZrOCl_2{\cdot}8H_2O$ and 0.08 mmol (34 mg) glycolipid in 3.5 mL (45 mmol) DMF, and the resulting mixture was incubated in an oven at 40 °C for 24 h.

Gels purification. After synthesis, the obtained gels were washed twice with DMF and thrice with ethanol. In each step, fresh solvent was added so that the total gel volume was expanded to double that of the as-synthesized gel. Using a vortex mixer, the gels were homogenized with the fresh solvent, after which the expanded gels were allowed to rest overnight at 120 °C for DMF-exchanged gels, and 60 °C for ethanol-exchanged gels. Subsequently, the gels were centrifuged, after which the supernatant solution was decanted. Following the final washing step, the volume of the gel was adjusted again by addition of fresh solvent, to achieve a volume equal to that of the as-synthesized gel.

Gel synthesis inside NMR tubes. 1 mL of the precursors mixture was prepared in DMF for each case (UiOG1, UiOG2 or Glyco@UiOG2), after which 120 µL of methanol-d4 was added. The resulting mixture was placed in a NMR tube and immediately inserted into the NMR instrument (previously heated at 40 ºC) to start the in situ gel formation measurements.

Synthesis of UiO-66 nanoparticles. Microcrystalline UiO-66 nanoparticles were prepared by dissolving 0.5 g (3 mmol) H_2BDC and 0.156 g (0.67 mmol) $ZrCl_4$ in 40 mL DMF, followed by addition of 20 mL HAc, and heating the mixture at 100 ºC for 1 h under stirring. After cooling to room temperature, the particles were collected by centrifugation (13,000 rpm, 5 min), washed twice with fresh DMF and 3 times more with MeOH.

Table S1. Optimized conditions for the synthesis of UiO-66 gels at 40 ºC.

Sample	H_2BDC (mmol)	$ZrOCl_2{\cdot}8H_2O$ (mmol)	DMF (mmol)	HAc (mmol)	Glyco (mmol)
UiOG1	1.2	0.8	45	2.8	-
UiOG2	1.2	0.8	45	-	-
Glyco@UiOG2	1.2	0.8	45	-	0.08

Maximum theoretical glycolipid loading. Assuming complete consumption of $ZrOCl_2·8H_2O$ (the limiting reactant), 0.8 mmol of $ZrOCl_2·8H_2O$ should produce 0.133 mmol of UiO-gel. The amount of glycolipid added is 0.08 mmol, which corresponds to 34 mg. Considering that the molecular formula of UiO-gels is the same as that of UiO-66 (i.e., $C_{48}H_{28}O_{30}Zr_6^{+4}$), with a molecular weight of 1632.0 g/mol, 0.133 mmol of UiO-gel corresponds to 217 mg. Therefore, the theoretical percentage of the glycolipid loaded in the gel, expressed as loading capacity, is 13.5%, as calculated using the following equation:

$$\mathrm{LC}\ (wt\%) = \frac{mg\ Glyco_{\mathrm{loaded}}}{mg\ Glyco@UiOG2} x\ 100$$

S3. Additional structural characterization of gels

SEM-EDX. To evaluate the homogeneity of the metal distribution within the gels, EDX mapping analysis was performed (Figure S1), revealing a uniform elemental distribution of Zr throughout the samples.

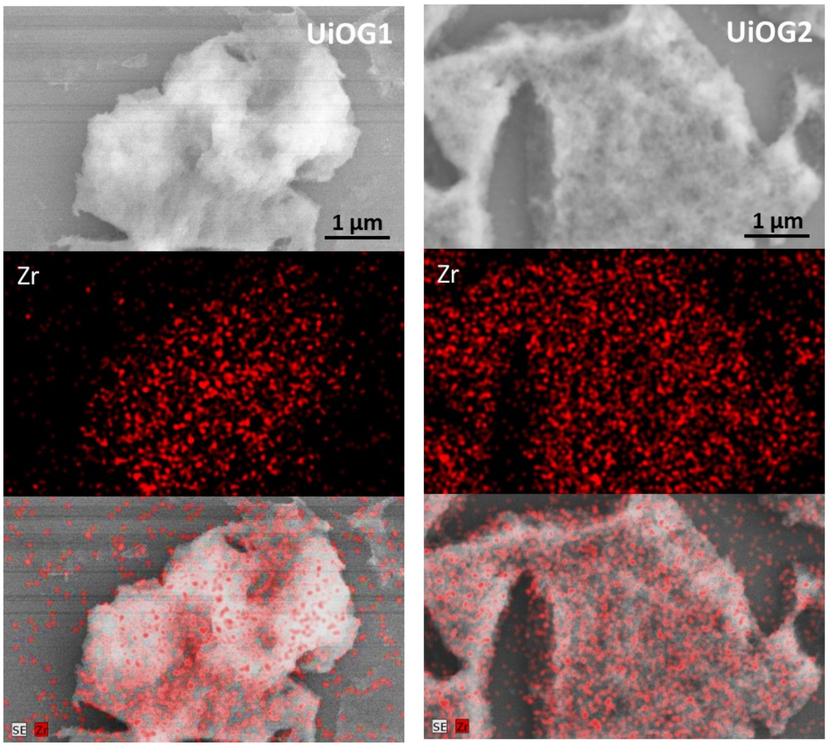

Figure S1. SEM micrographs and EDX elemental mapping of UiOG1 (left) and UiOG2 (right), recorded at 5 kV for 10 min. Zr distribution is shown in red.

XRD. To evaluate the homogeneity of the gels, a µ-diffraction mapping analysis was performed. The gel sample was deposited on a silicon zero-background holder (Figure S2A), and 2D-XRD patterns were collected from five distinct points across the sample surface (A-E) using a 2 mm collimator, covering a representative sample area (15.7 mm2) (Figure S2B). The resulting diffractograms from the different zones does not exhibit significant variations in peak position or breadth (Figure S2C). Moreover, the Debye rings in all 2D-diffractograms remain continuous and uniform in intensity, with no evidence of localized crystalline "hot spots" or significant variations in the amorphous background (Figure S2D).

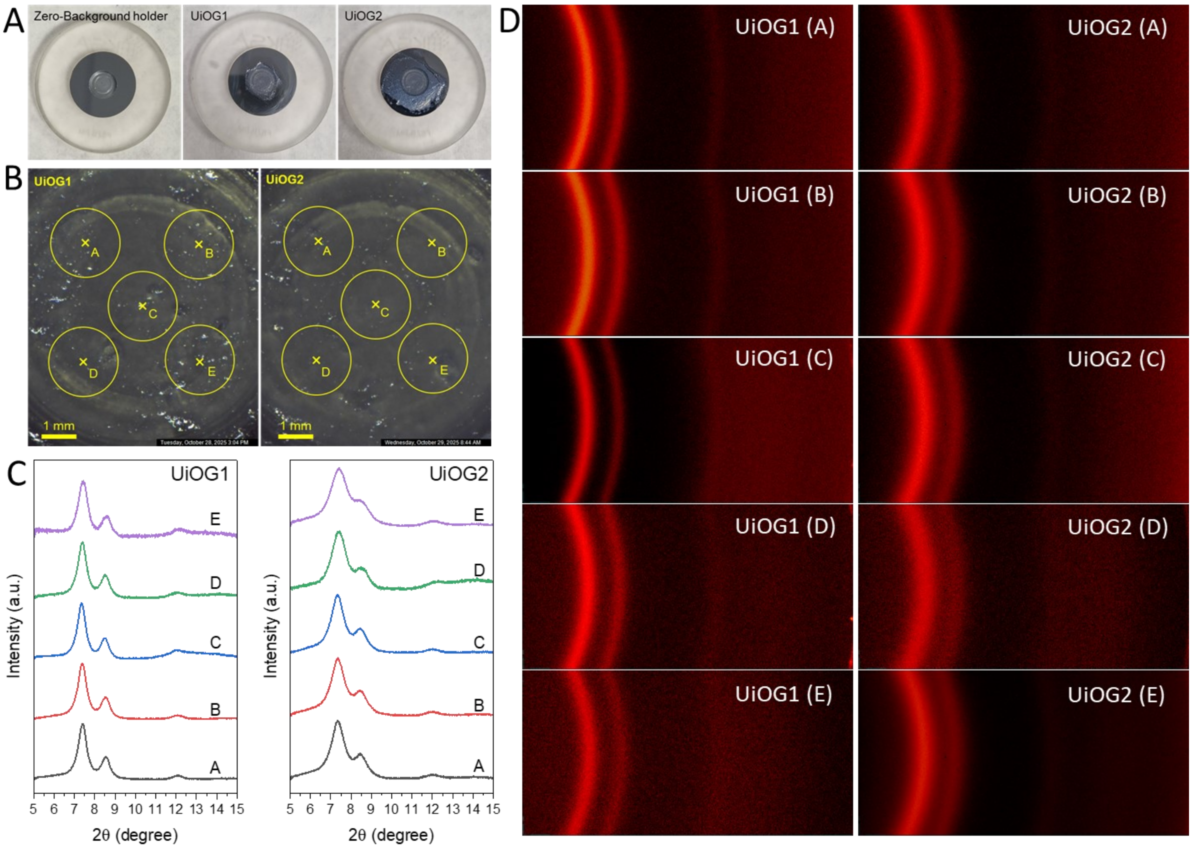

Figure S2. (A) Gel samples deposited on a silicon zero-background holder. (B) Regions of interest (A-E) analyzed by 2D-μ-XRD. (C) 1D diffractograms and (D) 2D diffractogram obtained from the five measurement points of the gel samples.

Furthermore, the crystallinity index (CI) was obtained for each of the analyzed regions. The CI is defined as the ratio of the crystalline domain (Icrystalline) and the total domain (crystalline and amorphous phases) of a material ($I_{crystalline}$ + $I_{amorphous}$) and is calculated by the following expression.

$$CI\ (\%) = \frac{I_{crystalline}}{I_{crystalline} + I_{amorphous}} \times 100$$

The CI of UiOG1 and UiOG2 are shown in Table S2. The calculated values show a very narrow distribution, remaining constant throughout the sample, confirming that the amorphous-to-crystalline ratio does not vary significantly across the gel.

Table S2. Crystalline Index (CI) distribution across different regions (A-E) of UiOG1 and UiOG2 gels.

region	UiOG1	UiOG2
A	68.1	65.3
B	72.1	64.5
C	59.9	71.1
D	79.5	50.9
E	57.3	58.6
Average	67.4 ± 8.1	62.1 ± 6.9

This consistency confirms that the UiO-66 crystalline phase is uniformly distributed throughout the gel matrix, with no evidence of phase separation or localized amorphous domains, thus supporting the conclusion that the gel is a homogeneous material composed of a continuous network of nanocrystals.

FT-IR. The coordination between Zr_6 clusters and the carboxylate ligands was confirmed by FTIR (Figure S3). H_2BDC ligands showed characteristic C=O (1685 cm^{-1}) and C–O (1370 cm^{-1}) stretches, which disappeared in the UiO-66 spectrum, giving rise to new asymmetric and symmetric carboxylate bands at 1570 and 1400 cm^{-1}. Bands at 745 and 659 cm^{-1} correspond to Zr–O vibrations, confirming ligand coordination. The FTIR spectra of the gels closely resembled that of the free ligand, attributable to excess uncoordinated BDC trapped on the gel network during gelation. This unbound BDC dominates the carboxylic and aromatic regions, making the overall spectrum similar to the pure ligand. Incorporation of the glycolipid was confirmed by the appearance of its characteristic IR bands in Glyco@UiOG2 (Figure 2A in the main manuscript).

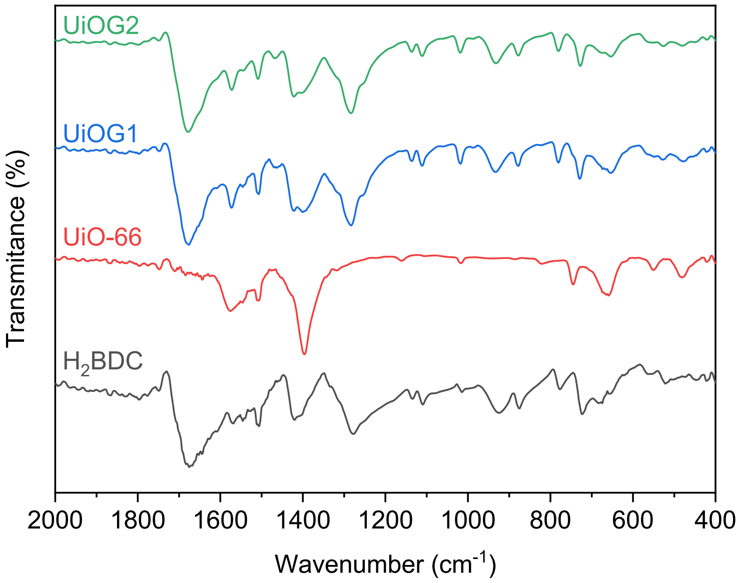

Figure S3. FT-IR spectra of the two studied gels (UiOG1 and UiOG2) compared with those of microcrystalline UiO-66 nanoparticles and the H_2BDC linker.

TGA. The TGA curves of UiO-66, UiOG1, and UiOG2 revealed distinct weight-loss events corresponding to different thermal decomposition stages (Figure S2).

To quantify the structural composition of pristine UiO-66 and the gel-like samples, the Zr-to-linker molar ratios (n_{Zr}/n_{BDC}) were determined from the relative weight losses after subtracting the fraction of coordinated species (Figure S4). In an ideal 12-connected UiO-66, the theoretical n_{Zr}/n_{linker} ratio is 1 [2]. TGA analysis showed slight deviations from this value: 1.03 for UiO-66, 1.07 for UiOG1, and 1.05 for UiOG2, indicating minor linker deficiencies (Table S3). The corresponding deficiencies were estimated as 2.9 % for UiO-66, 6.5 % for UiOG1, and 5.4 % for UiOG2, consistent with the higher defect density in UiOG1 caused by the acid modulator. These calculations assume complete decomposition of the organic matter, with the residual mass attributed solely to ZrO_2. The n_{Zr}/n_{linker} ratios were derived from the mass percentages of ZrO_2 and organic linker as follows:

$$n_{Zr} = n_{ZrO_2} = \frac{\%ZrO_2 \cdot m_{total}}{Mw(ZrO_2)}$$

$$n_{BDC} = \frac{\%BDC \cdot m_{total}}{Mw(BDC)}$$

$$\frac{n_{Zr}}{n_{BDC}} = \frac{\frac{\%ZrO_2 \cdot m_{total}}{Mw(ZrO_2)}}{\frac{\%BDC \cdot m_{total}}{Mw(BDC)}} = \frac{\%ZrO_2 \cdot Mw(BDC)}{\%BDC \cdot Mw(ZrO_2)}$$

where $\%ZrO_2$ is percentage of residue at high temperature (>600 °C), %BDC is the estimated mass loss due to combustion of the organic linker (500–600 °C), m_{total} is the total mass of the sample used for the analysis and $Mw(ZrO_2)$ and Mw(BDC) are the molecular weights of ZrO_2 and BDC, respectively.

From these ratios, the linker deficiency (relative to the ideal UiO-66 formula $Zr_6O_4(OH)_4(BDC)_6$) for each sample was estimated (shown in Table S3) by using the following equation:

$$Defectiviy = 1 - \left(\frac{n_{Zr}}{n_{BDC}}\right)^{-1}$$

Table S3. Calculation of the linker deficiency for the microcrystalline UiO-66 and UiO-gels.

Sample	$\%ZrO_2$	%BDC	n_{Zr}/n_{BDC}	Defectivity × 100 (%)
UiO-66	39.3	48.2	1.03	2.80
UiOG1	15.6	23.8	1.07	6.54
UiOG2	18.7	19.5	1.05	5.39

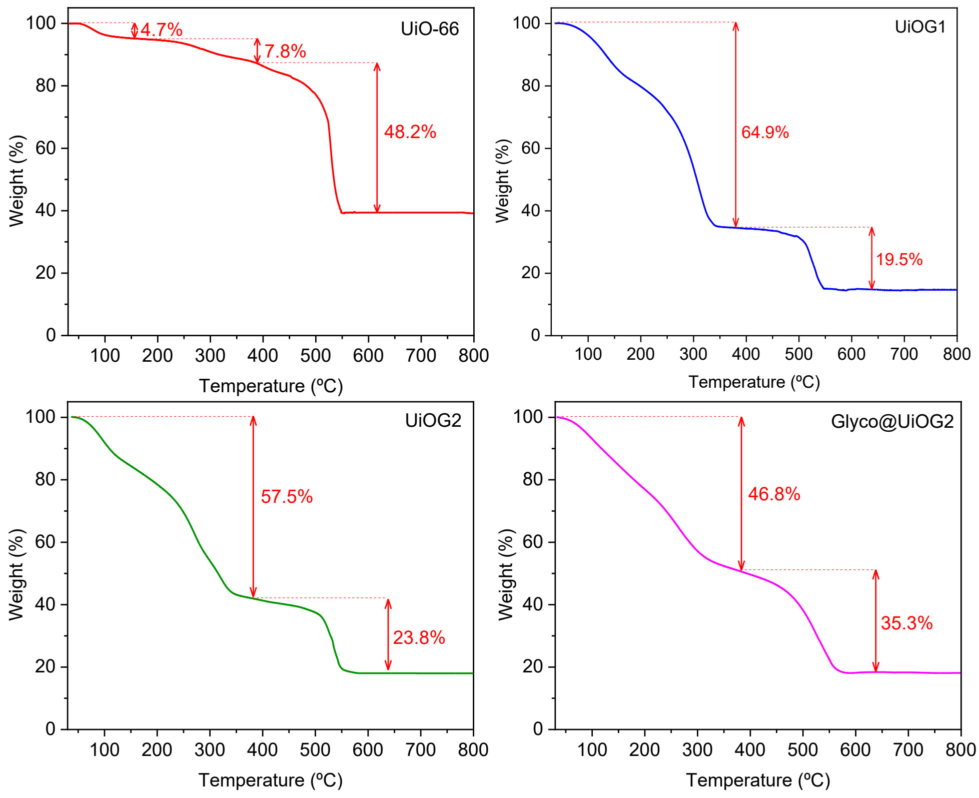

Figure S4. TGA profiles of the samples, displaying the weight-loss percentage as a function of temperature.

The incorporation of the glycolipid into the UiOG2 framework was evaluated based on the relative weight loss in the TGA of Glyco@UiOG2. Assuming that the Zr/BDC molar ratio remains unaltered upon glycolipid encapsulation within the gel-like structure, the excess weight loss (beyond that attributable to ZrO_2 and BDC) can be attributed to the glycolipid content. Under this assumption, the mass percentage of glycolipid was estimated to be 12.4%, which is in good agreement with the theoretical value of 13.5 %wt (i.e., amount added to the precursors mixture during the gel synthesis).

$$\left(\frac{n_{Zr}}{n_{BDC}}\right)_{UiOG2} = 1.05 = \frac{\%ZrO_2 \cdot Mw(BDC)}{\%BDC \cdot Mw(ZrO_2)} \rightarrow \%BDC \ = \ 22.9\%$$

$$\%Glyco = 100 - (\%solvent\ and\ coordinated\ species + \%BDC + \%ZrO_2)$$
$$= 100 - 46.8 - 22.9 - 17.9 = 12.4\%$$

N_2 isotherms. The isotherms of the gels revealed significant diffrences in pporosity with respect to microcrystalline UiO-66 nanoparticles (Figure S5, Table S4).

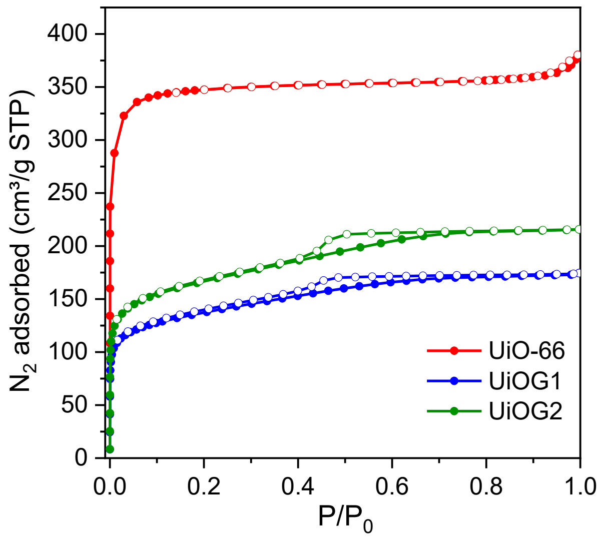

Figure S5. N_2 adsorption/desorption isotherms (77 K) for gels (UiOG1 and UiOG2) and the microcrystalline UiO-66 nanoparticles for comparison.

Table S4. Textural properties of the two studied gels (UiOG1 and UiOG2) compared with those of microcrystalline UiO-66 nanoparticles.

Sample	S_{BET} (m^2/g)	S_{micro} (m^2/g)	S_{ext} (m^2/g)	V_{total} (cm^3/g)	V_{micro} (cm^3/g)	V_{meso} (cm^3/g)
UiO-66	1168	1049	119	0.57	0.48	0.09
UiOG1	502	288	214	0.28	0.13	0.15
UiOG2	585	319	266	0.35	0.16	0.19

Rheology. The flow behaviour of the different gels as a function of shear rate were fitted using the Cross model (R^2>0.99) and their fitting parameters are in Table S5. This model is defined by:

$$\eta = \eta_\infty + \frac{\eta_0 - \eta_\infty}{1 + (k \cdot \dot{\gamma})^m}$$

where η_0 is the zero-shear viscosity, k is the inverse of the consistency index, m is the shear-thinning index, and $\dot{\gamma}$ is the shear rate.

Table S5. Cross model fitting parameters for the flow curves for the different prepared gels.

Sample	η_∞ (Pa·s)	η_0 (Pa·s)	k (s)	m	R^2
UiOG1	$2.66 \cdot 10^{-11}$	4341	19.95	1.98	0.999
UiOG2	$7.08 \cdot 10^{-8}$	23823	21.55	1.88	0.999
Glyco@UiOG2	$5.83 \cdot 10^{-10}$	13976	21.05	1.88	0.999

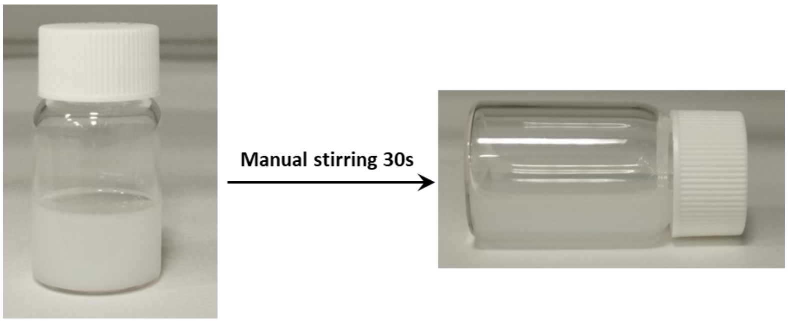

Figure S6. Transition from "non-flowing" to "flowing" state upon mechanical stirring (manual stirring for 30 s). Initially, the UiOG2 gel is "non-flowing," but after stirring they convert into "flowing" gels. When left undisturbed for several hours, they revert to their initial non-fluid state.

S4. Additional NMR data of gel formation process

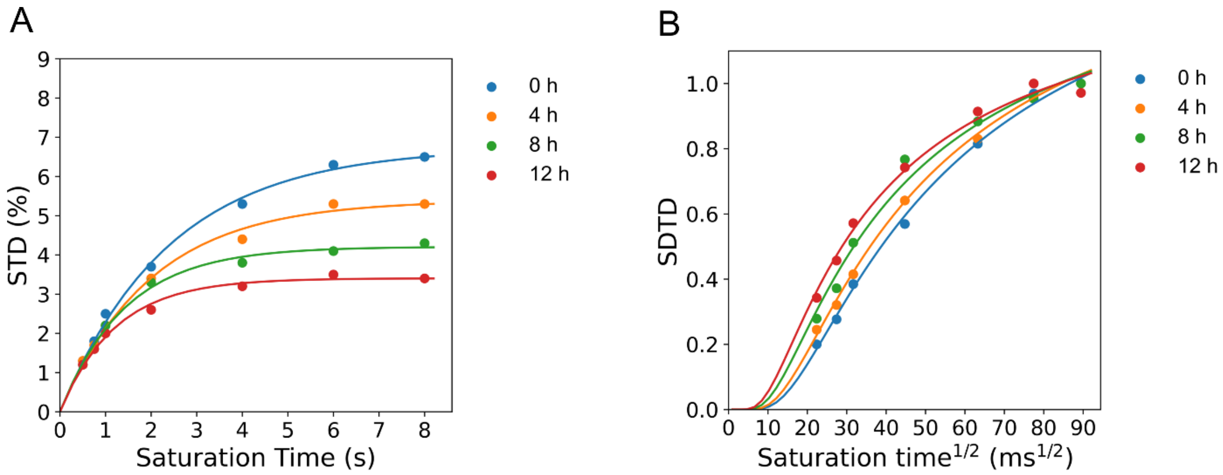

Figure S7. STD (A) and SDTD (B) build-up curves monitored during the in situ formation of Glyco@UiOG2 gel, using a 500 MHz NMR spectrometer, over a period of 12 h.

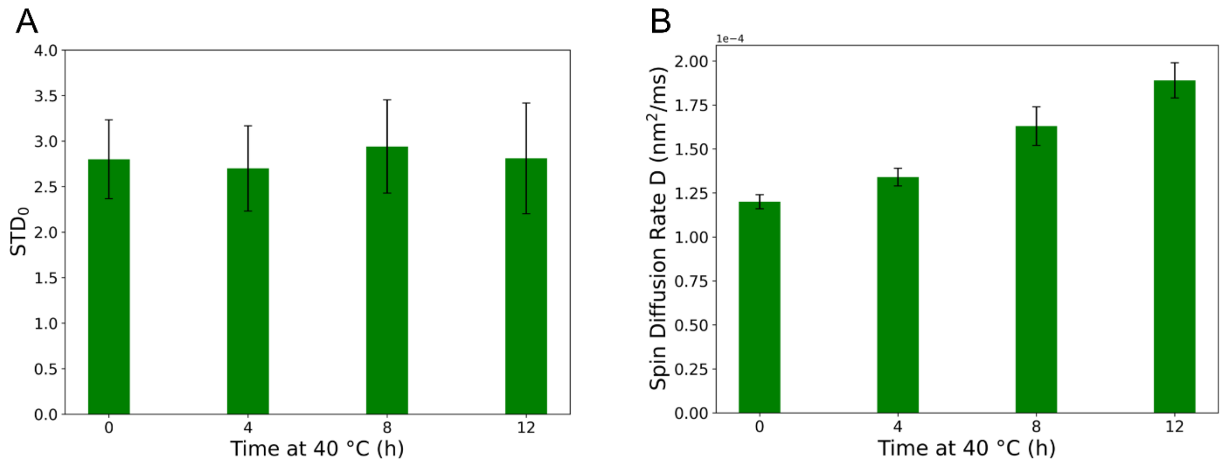

Figure S8. Evolution of the STD_0 (A) and spin diffusion rate D (B) of water obtained during the in situ gelation of Glyco@UiOG2 (green bars), using a 500 MHz NMR spectrometer, over a period of 12 h. The uncertainties associated to the determination of STD_0 (A) and D (B) are shown as error bars.

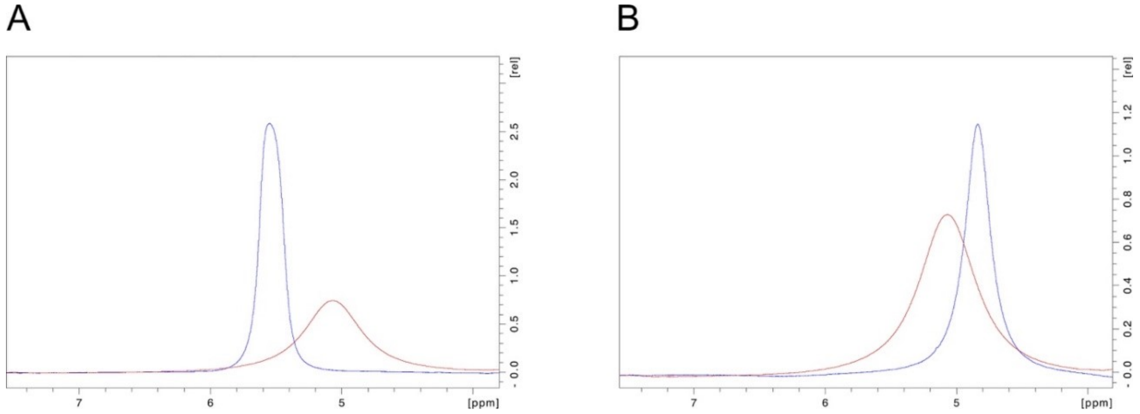

Figure S9. Overlay of the water region of the ^{1}H NMR spectra of UiOG1 (A) and UiOG2 (B) gels acquired at the beginning (time 0) of the in situ gel formation (blue spectra; prenucleation stage) and after 18 h at 40 °C (red spectra; gel-like state).

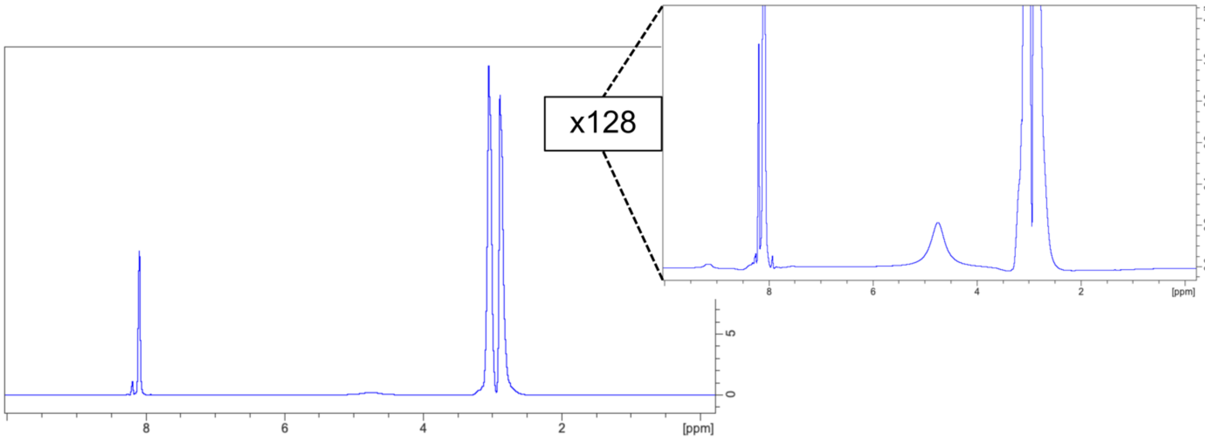

Figure S10. ^{1}H NMR spectrum of UiOG2 showing the DMF peaks around 3 and 8.1 ppm. The zoomed spectrum shows the water peak around 4.7 ppm.

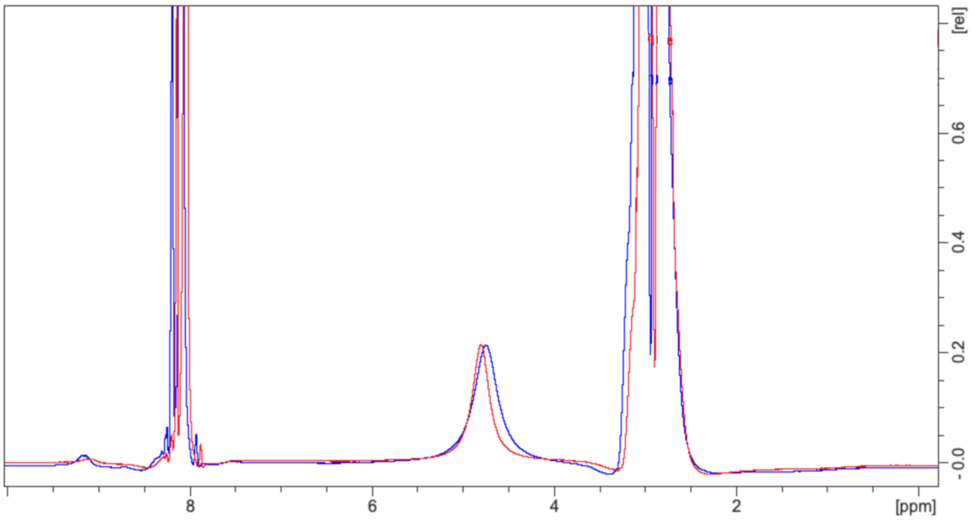

Figure S11. Overlay of the ^{1}H NMR spectra of UiOG2 at time 0 (blue) and after 12 h at 40 °C (red).

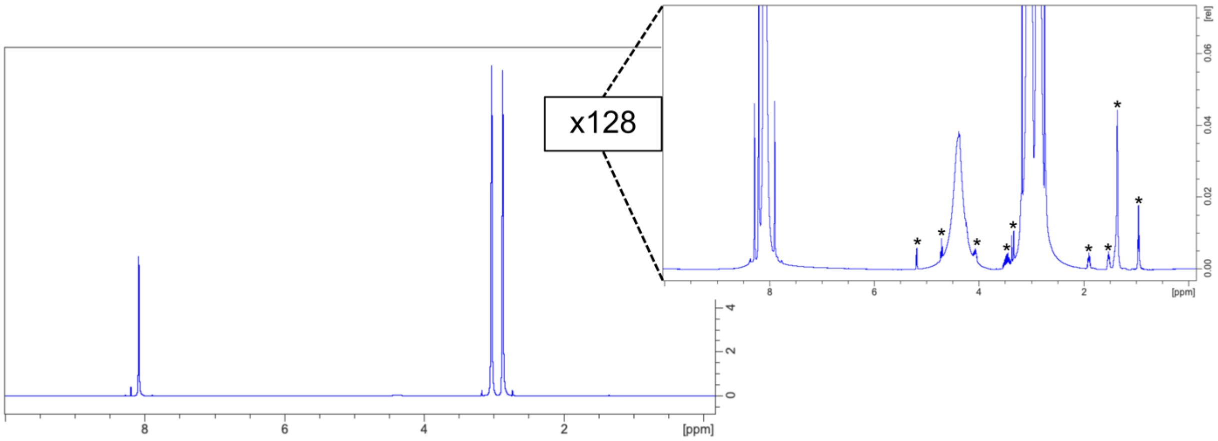

Figure S12. ^{1}H NMR spectrum of Glyco@UiOG2 showing the DMF peaks around 3 and 8.1 ppm. The zoomed spectrum shows the water peak around 4.4 ppm and the signals of the glycolipid (indicated with an asterisk on top).

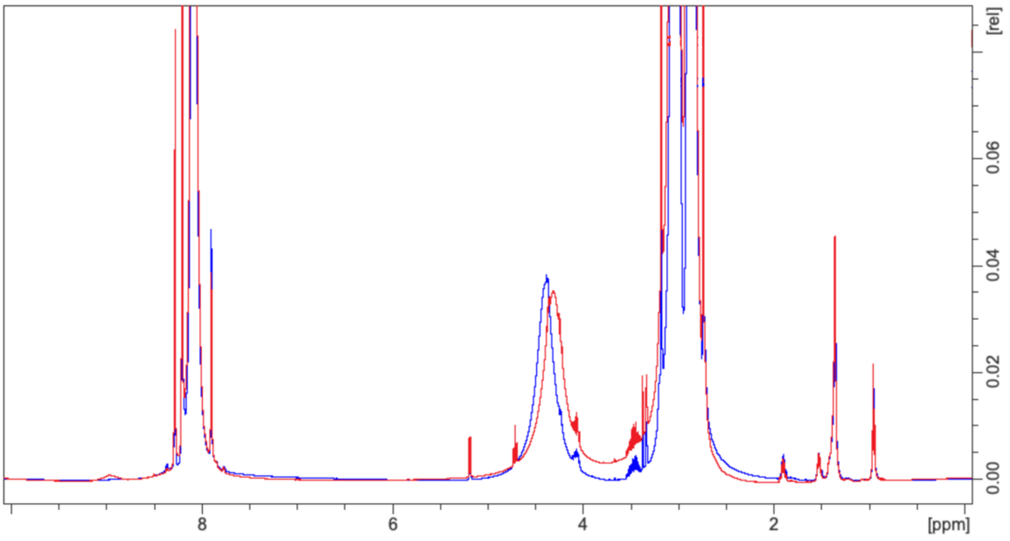

Figure S13. Overlay of the ^{1}H NMR spectra of Glyco@UiOG2 at time 0 (blue) and after 12 h at 40 °C (red).

S5. References

[1] E. M. Sánchez-Fernández, V. Gómez-Pérez, R. García-Hernández, J. M. García Fernández, G.B. Plata, J.M. Padrón, C. Ortiz Mellet, S. Castanys, F. Gamarro, “Antileishmanial activity of sp2-iminosugar derivatives” *RSC Adv.* **2015**, *5*, 21812.

[2] D. K. Sannes, S. Øien-Ødegaard, E. Aunan, A. Nova, U. Olsbye, “Quantification of linker defects in UiO-type metal–organic frameworks” *Chem. Mater.* **2023**, *35*, 3793-3800.
